# Evaluation of Trauma-Induced Coagulopathy by Systematic Insights Into Pathophysiology and Advances in Emergency Resuscitation

**DOI:** 10.7759/cureus.83839

**Published:** 2025-05-10

**Authors:** Noor Ul Ain Rashid, Ahmad Abdullah Nasir, Bilal Fattani, Madeeha Minhas, Seemi Tanvir, Soobia Pathan, FNU Barkha, Pirya Nangdev, Aneesa Khalid

**Affiliations:** 1 General Surgery, Akhtar Saeed Trust Hospital, Lahore, PAK; 2 General Surgery, General Medicine, Anesthesiology and Psychiatry, Akhtar Saeed Medical and Dental College, Lahore, PAK; 3 Medicine, Jinnah Medical and Dental College, Karachi, PAK; 4 Health Sciences, King Saud Bin Abdulaziz University for Health Sciences, Jeddah, SAU; 5 Pathology, Margalla Institute of Health Sciences, Rawalpindi, PAK; 6 Pharmacology and Therapeutics, Liaquat Institute of Medical and Health Sciences, Thatta, PAK; 7 Medicine and Surgery, Chandka Medical College, Larkana, PAK; 8 Anatomy, Bilawal Medical College, Liaquat University of Medical and Health Sciences, Jamshoro, PAK; 9 Pathology, University of Health Sciences, Lahore, PAK; 10 Biomedical Sciences, COMSATS (Commission on Science and Technology for Sustainable Development in the South) University Islamabad, Islamabad, PAK; 11 Molecular Pathology and Genetics, University of the Punjab, Lahore, PAK

**Keywords:** fibrinolysis, hemostasis, meta-analysis, resuscitation, trauma-induced coagulopathy, viscoelastic testing

## Abstract

Trauma-induced coagulopathy (TIC) exists as a fatal complication that develops from severe injuries and substantially increases patient mortality. Effective knowledge of TIC pathophysiology, together with optimized resuscitation strategies, is fundamental for enhancing patient outcomes. This review system examined TIC mechanisms through an analysis of present-day intervention methods. This systematic research with meta-analysis covered PubMed, Scopus, and Web of Science, together with Google Scholar databases, to review TIC pathophysiology and resuscitation practices. The inclusion of observational and experimental designs occurred by using predetermined selection criteria. Two independent researchers collected data from studies that received quality assessments through the Newcastle-Ottawa Scale (version 2011), along with the Cochrane Risk of Bias Tool (version 2) evaluation. The GRADE (Grading of Recommendations Assessment, Development, and Evaluation) approach served as the method for evaluating evidence certainty. The analyzed studies amounted to 11 after meeting the established criteria. Three major mechanisms led to TIC development, including disrupted fibrinolysis function, impaired platelet functioning, and damaged vessel walls. Six studies submitted for meta-analysis demonstrated that coagulation abnormalities, including hypofibrinogenemia and elevated activated protein C, result in adverse outcomes with a pooled HR of 6.3 (95% CI: 3.04-13.05, p < 0.05). The compound variation (I² = 69%) across data points appeared from differences between measurements and experimental conditions used in the studies. This review presents fibrinogen together with thrombin and activated protein C as diagnostic markers for TIC while supporting the use of protocol-based resuscitation. Future research requires several hospitals to participate in trials to establish new treatment protocols and establish uniform biomarker testing procedures in clinical settings.

## Introduction and background

Trauma-induced coagulopathy (TIC) is a life-threatening condition that contributes significantly to the rise in global trauma-related mortality rates [1]. It is frequently encountered in the emergency setting and poses major challenges during early resuscitation. Despite current resuscitation and trauma care efforts, managing severe TIC cases remains largely ineffective [2]. The condition’s complex pathophysiology, including excessive fibrinolysis, platelet dysfunction, and endothelial damage, requires timely recognition and appropriate interventions. However, trauma patients often undergo inadequate assessment of coagulation abnormalities, as clinical observations and standard laboratory tests are insufficient [3].

TIC progresses through three pathological processes, including excessive fibrinolysis, platelet dysfunction, and endothelial tissue damage. This leads to a vicious cycle where uncontrolled bleeding and coagulopathy exacerbate each other, increasing patient morbidity [4]. Crosslinking with emergency resuscitation, conventional coagulation testing methods fall short in detecting the dynamic development of TIC, which results in delayed medical intervention. Moreover, current treatment standards are inconsistent, contributing to varied clinical outcomes and highlighting the need for optimal resuscitation strategies. Medical professionals often struggle to balance hemostasis and thrombosis, underscoring the need for improved protocols in the administration of blood products and antifibrinolytic therapies, as well as in the management of complications [5].

A meta-analysis of observational data allows the authors to develop better evidence about the research topic. The modern research on TIC pathogenesis has allowed scientists to better understand fibrinolysis, as well as platelet dysfunction and endothelial injury mechanisms [6]. Patient resuscitation outcomes should improve due to new blood transfusion protocols, administration of hemostatic agents, and rapid intervention strategies. The review establishes diagnostic methods and treatment approaches that lead to better recovery outcomes and survival prospects of patients through examination of TIC clinical manifestations and molecular signals.

## Review

Methodology

The review followed the Preferred Reporting Items for Systematic Reviews and Meta-Analyses (PRISMA) guidelines (2020) [7] to provide a transparent assessment of pathophysiological aspects and resuscitation methods appearing in TIC. The selection process used predefined eligibility standards to evaluate both the research design and essential clinical outcomes from TIC, including survival statistics, treatment effectiveness, and thrombus control results. Multiple electronic databases, such as PubMed, Scopus, Web of Science, and Google Scholar, were used to conduct the systematic search. Research studies from 2019 to 2025 were included in the review that exclusively used English as the publication language. The research plan contained search terms that integrated "trauma-induced coagulopathy" along with "pathophysiology", "resuscitation", "emergency care", and "hemostasis". Boolean operators joined these keywords, when necessary, while filters helped to retrieve the most suitable results. The study checked the relevance of all articles through title examination, along with abstract and full text evaluation processes.

Two researchers independently conducted evaluations based on the pre-determined selection standards for potential studies. When discrepancies arose during screening, both experts discussed the findings while requesting guidance from a third expert. Each reviewer accomplished data extraction independently through standardized forms, which covered study characteristics along with TIC pathophysiology and resuscitation strategies, and clinical outcomes. Each study's potential bias risk was evaluated through suitable assessment methods consisting of the Newcastle-Ottawa Scale (version 2011) for cohort studies and the Cochrane Risk of Bias Tool (version 2) for randomized controlled trials (RCTs).

The evaluators utilized the GRADE (Grading of Recommendations Assessment, Development, and Evaluation) approach to assess evidence quality by examining study designs together with risk of bias, along with findings consistency. The intense methodology brought a comprehensive review of TIC management as well as resuscitation approaches. A meta-analysis was performed specifically to analyze observational study hazard rates from 2019 to 2024. This investigation selected observational studies together with prospective and retrospective cohort research and clinical assessments, which monitored target outcomes using hazard rate evaluations involving factor XIII (FXIII) depletion and fibrinogen depletion, and elevated international normalized ratio (INR)/prothrombin time (PT) results. Research testing for three specific criteria would automatically result in exclusion from further review: RCT status, case reports alongside reviews, and the absence of control groups. This research conducted a random-effects analysis that produced inverse variance-weighted effect sizes with 95% confidence intervals for quantitative synthesis. Sequential exclusion methods were used for sensitivity analysis, and the I² statistics measured report heterogeneity [8]. The analysis checked statistical significance with a p-value set below 0.05.

Results

The review procedure led to the selection of 11 publications from a total of 102 analyzed through multiple databases. The research examined coagulopathy and coagulation dysfunction in trauma patients from multiple points during the aftermath. The available literature consisted of one case report, together with two RCTs and five prospective observational studies, along with two retrospective studies having participant counts from one to 503 and a median of 160 people.

**Figure 1:**
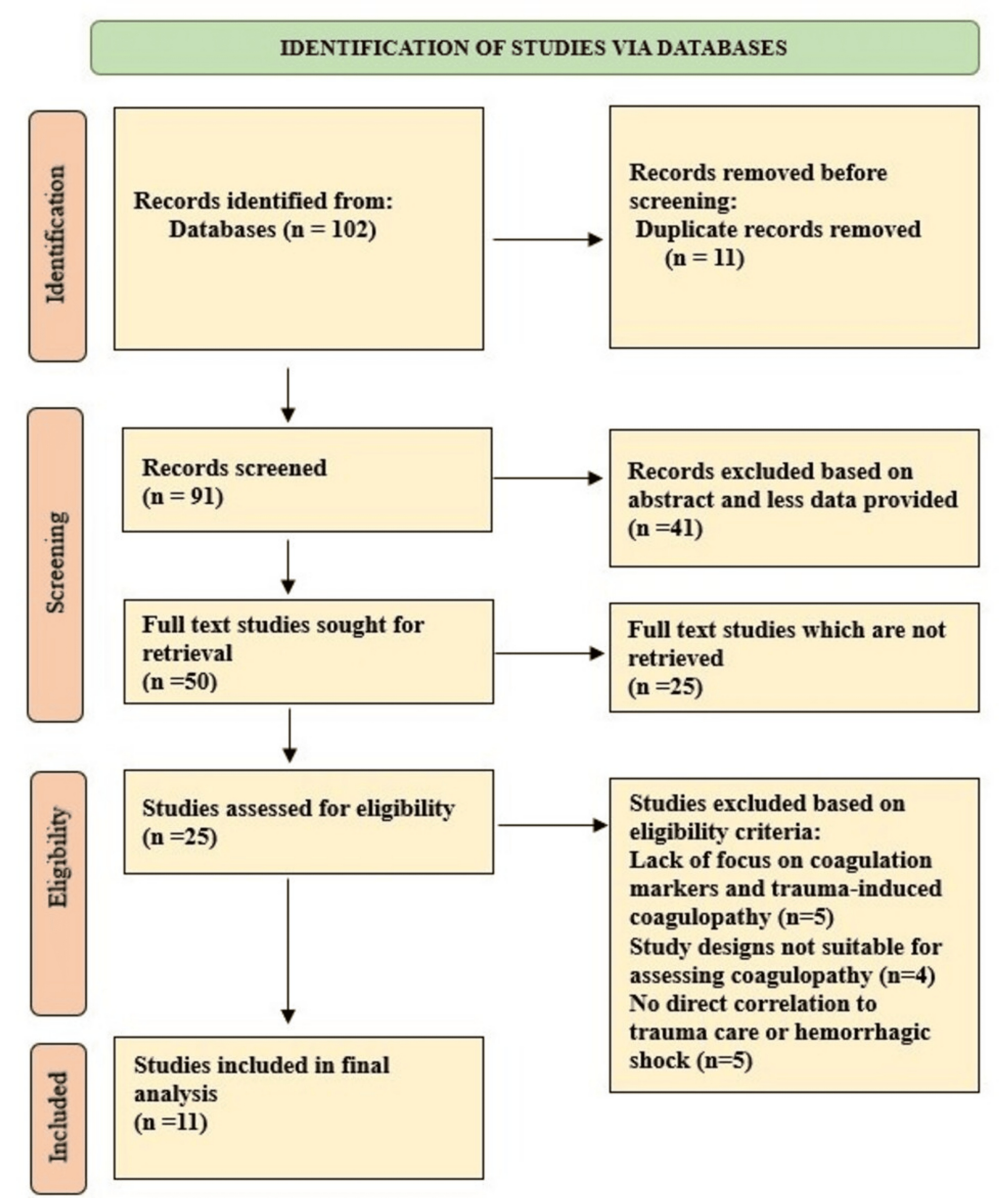
Filtration process of gathered studies till final selection of studies using the PRISMA guidelines. PRISMA: Preferred Reporting Items for Systematic Reviews and Meta-Analyses.

Different types of traumas consist of severe traumatic brain injury and burns, together with several other forms of injury. Research results revealed separate coagulopathy states, including severe coagulopathy and hyperfibrinolysis, while fibrinogen levels, together with other coagulation factors, predicted patient recovery. Figure 1 illustrates the complete process of study selection, as depicted in the image.

The investigative studies analyzed blood clotting elements in traumatically injured patients to assess TIC along with coagulation irregularities and their associated consequences. Different types of traumatic injuries, including severe brain injuries, burns, and blunt trauma, were included in reviewed research studies, which used multiple types of study designs from prospective observational studies to RCTs and case reports. The research demonstrated that fibrinogen and FXIII, together with thrombin and activated protein C (APC), provided important information to anticipate transfusion requirements along with mortality risks and unfavorable hemodynamic responses. A summary of these data can be found in Table 1, which reveals how multiple elements in the coagulation system work together to affect trauma patients.

**Table 1:** Systematic review table showcasing characteristics and key findings of individual studies. ISS = Injury Severity Score; SHINE = severe hemorrhagic injury and neutrophil evaluation; APC = activated protein C; CT = computed tomography; FM = fibrin monomers; PHI = progressive hemorrhagic injury; ED = emergency department; VICC = venom-induced consumptive coagulopathy; TIC = trauma-induced coagulopathy; BE = base excess; ICU = intensive care unit; TBI = traumatic brain injury; VHA = viscoelastic hemostasis assay; CCT = conventional coagulation test; A5/A10 = thromboelastography amplitudes at 5 and 10 minutes; MA = maximum amplitude; FXIII = factor XIII; INR = international normalized ratio.

Author & year	Sample size	Study design	Confounders	Outcomes measured	Key findings
Johansson et al. (2024) [9]	313	Multicenter observational	Injury Severity Score (ISS)	SHINE phenotypes, transfusion requirements, mortality, and coagulation factors	TIC is present in phenotype 2, leading to higher transfusion needs and mortality
Caspers et al. (2022) [10]	73	Prospective observational	Injury Severity Score (ISS), trauma mechanism	Plasma coagulation markers (thrombin, APC levels), clinical course, patient outcome	Higher levels of thrombin and APC are linked to worse outcomes
Esnault et al. (2020) [11]	160	Prospective observational	Age, injury severity, coagulopathy, initial CT scan findings	Fibrin monomers (FM) levels, progressive hemorrhagic injury (PHI), functional neurologic outcome at 6 months	Higher levels of FM are linked to worse neurologic outcomes
Bodnar et al. (2024) [12]	216	Prospective observational	Pre-hospital time, blunt trauma, crystalloids volume, systolic blood pressure, pre-hospital temperature	Mortality, ED transfusion, ISS, coagulation derangement	Hypofibrinogenemia is linked to mortality and early transfusion
Moon et al. (2021) [13]	119	Retrospective study	Time to antivenom administration, baseline coagulation parameters, severity of VICC	Development of VICC, clotting factors, blood transfusion, time course of D-dimer, and fibrinogen	Factors II and X are reduced in complete VICC; fibrinogen is an early marker
George et al. (2022) [14]	68	Prospective multicenter, randomized trial	Trauma-induced coagulopathy (TIC), transfusion therapy, fibrinogen concentrate vs. cryoprecipitate	Time to fibrinogen replacement, clinical secondary outcomes, and feasibility outcomes	Early fibrinogen replacement in pediatric trauma patients may decrease complications
Savioli et al. (2020) [15]	503	Prospective, monocentric, observational	Trauma severity, gender, head trauma, abdominal trauma, injury severity score, shock index, lactate, acidemia, BE (base excess)	Hemotransfusion rate, hemodynamic instability, hospitalization rate, ICU admission, length of ICU stay, mortality, lactate, and acid-base imbalance	Trauma coagulopathy patients required more hemotransfusion
Baksaas-Aasen et al. (2021) [16]	396	Randomized controlled trial	Severe traumatic brain injury (TBI), age, and injury severity	Proportion of patients alive and free of massive transfusion at 24 hours, 28-day mortality, secondary outcomes including serious adverse events	VHA had a better outcome for 24-hour survival and freedom from massive transfusion
Vigstedt et al. (2022) [17]	187	Randomized controlled trial	Hemorrhagic shock, trauma severity, treatment allocation (CCT vs. VHA)	Sensitivity and specificity of A5 and A10 for predicting low MA	Early measurements (A5 and A10) from TEG® 6s predict low MA in trauma patients
Guilabert et al. (2024) [18]	21	Prospective observational pilot study	Surgical bleeding, fluid resuscitation	FXIII levels, coagulation factors, and coagulopathy	Significant decline in FXIII levels; coagulation factor levels decreased significantly 24 hours post-burn
Motono et al. (2024) [19]	1	Case report	Age, epilepsy, multiple fractures	Hemothorax progression, coagulopathy, hemostasis	Prolonged INR and low platelets; reoperation was required, and hemostatic materials were used.

The analyzed research articles examined trauma patients by studying coagulation markers, particularly regarding their connection to TIC and patient treatment results. Johansson et al. (2024), alongside Caspers et al. (2022), established that particular TIC phenotypes correlate with critical coagulation problems while generating adverse clinical results [9,10]. Johansson et al. (2024) described unique SHINE (severe hemorrhagic injury and neutrophil evaluation) categories while revealing that phenotype 2 patients developed severe coagulopathy and hyperfibrinolysis that required extra transfusions and caused a 39% death rate [9]. Research by Caspers et al. (2022) confirmed that elevated thrombin levels combined with APC would result in complicated recovery and inferior outcomes for patients with severe injuries [10].

The studies conducted by Esnault et al. (2020), together with Bodnar et al. (2024), showcased that detecting coagulation markers early can help predict the progression, along with mortality outcomes in patients [11,12]. According to Moon et al. (2021), fibrinogen functions as a key indicator of TIC, but George et al. (2022) demonstrated that pediatric trauma patients benefited from prompt fibrinogen replacement through improved survival rates, together with decreased transfusion needs and fewer complications [13,14].

This study paved the way for the evaluation of TIC and coagulation markers, emphasizing moderate to high levels of research bias. Controlled and confounding variables were managed based on assessments from the Newcastle-Ottawa Scale presented in Table 2. Evidence evaluations according to the GRADE system show that currently available data do not provide enough justification to establish specific coagulation markers as guiding tools for clinical decisions in trauma situations. Healthcare professionals need additional, extensive multicenter clinical trials to validate findings and establish definitive conclusions about these results.

**Table 2:** Risk of bias assessment of individual observational studies. Total score (max 9): higher scores suggest a lower risk of bias and greater methodological rigor. 7–9 stars: low risk of bias; 4–6: moderate risk of bias; <4: high risk of bias.

Study	Selection (max 4)	Comparability (max 2)	Outcome (max 3)	Total score (max 9)
Johansson et al. (2024) [9]	★★★★	★	★★★	8
Caspers et al. (2022) [10]	★★★★	★	★★★	8
Esnault et al. (2020) [11]	★★★★	★★	★★★	9
Bodnar et al. (2024) [12]	★★★★	★★	★★★	9
Moon et al. (2021) [13]	★★★	★★	★★★	8
Savioli et al. (2020) [15]	★★★	★★	★★	7
Guilabert et al. (2024) [18]	★★★	★★	★★	7
Motono et al. (2024) [19]	★★★	★★	★★	7

The assessment protocols in RCTs demonstrated inconsistent practices since data collection was incomplete and measurement procedures for outcomes were poorly defined for some studies, according to the Cochrane Risk of Bias Tool, as shown in Table 3.

**Table 3:** Risk of bias assessment of individual randomized controlled trials. "+" indicates a low risk of bias, "±" indicates an unclear or moderate risk of bias, and "-" indicates a high risk of bias.

Study	Sequence generation – selection bias	Allocation sequence concealment – selection bias	Blinding of participants and personnel – performance bias	Blinding of outcome assessment – detection bias	Incomplete outcome data	Selective outcome reporting	Other bias
George et al. (2022) [14]	+	+	±	±	+	+	+
Baksaas-Aasen et al. (2021) [16]	+	+	+	+	+	+	+
Vigstedt et al. (2022) [17]	+	+	+	+	+	+	+

**Figure 2:**
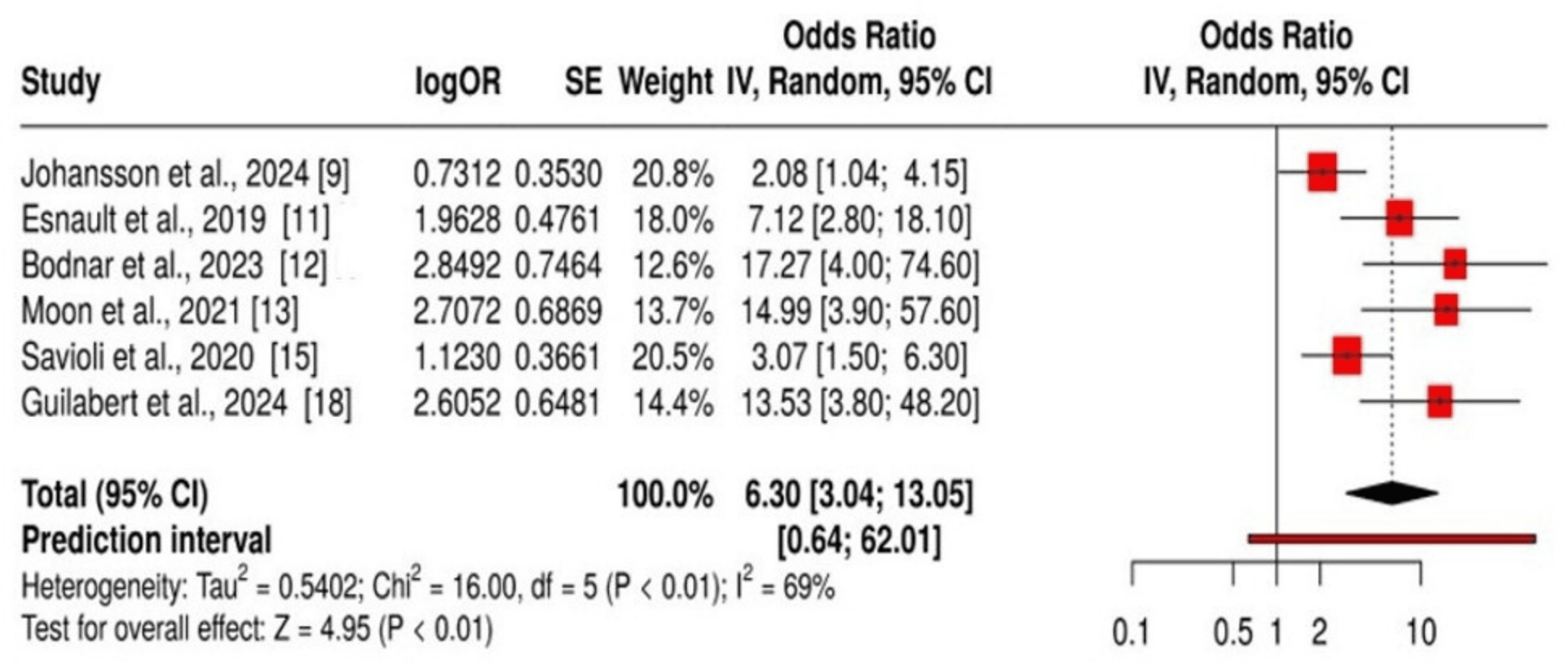
Forest plot exhibiting the association between fibrinogen, thrombin, and activated protein C markers with trauma-induced coagulopathy outcomes.

Analyses through heterogeneity testing showed significant differences existed between the included studies (p < 0.01) because the results did not match. The calculated I² statistic reached 69%, which suggested that 69% of the result variation stemmed from study differences rather than random measurement fluctuations. The moderate-to-high heterogeneity level indicates that studies presented different effect sizes because their populations used varying methodologies or uncontrolled dynamic variables, as shown in Figure 2.

Discussion

This systematic review and meta-analysis investigated the connection between coagulation markers and TIC to establish how biomarkers identify acute trauma patient severity and clinical outcomes. The review analyzed 11 main research studies together with six observational studies that demonstrated how fibrinogen, thrombin, and APC biomarkers can identify TIC and predict patient clinical outcomes. Research demonstrated that impaired coagulation leads to elevated mortality rates (pooled HR: 6.3; 95% CI: 3.04-13.05) and increased hospital stays and blood product transfusions [20]. Research has demonstrated that elevated biomarkers in traumatic patients are linked to coagulopathy, thus showing potential as early diagnosis indicators of TIC [21]. These biomarkers serve as vital indicators for risk assessment because they support the determination of patients who need immediate transfusion care alongside potential targeted therapeutic interventions [22].

This research has identified important biomarkers that could help physicians make treatment decisions. The proposed medical procedure of tracking fibrinogen levels lets healthcare providers determine which patients need fibrinogen concentrate treatment following traumatic injuries [23]. The assessment of thrombin generation and APC levels indicates coagulopathy severity as well as bleeding complications [24]. The use of these research findings poses multiple obstacles in implementing them for direct application in medical settings. Research heterogeneity exists at a high level (I² = 69%) because studies use different patient demographics and injury types alongside various biomarker measurement timings. The poor application of the results to large, diverse trauma populations exists because most studies used insufficient follow-up methods with small sample sizes [25].

The reported studies failed to establish consistent procedures for measuring and reporting coagulation marker values, which led to additional difficulties when interpreting their outcomes. The assessment of coagulation included studies that employed viscoelastic testing through rotational thromboelastometry (ROTEM) or thromboelastography (TEG), or they used traditional clotting assays for measurement [26,27]. Studies employing different laboratory diagnostic methods probably contribute to the discrepancy discovered in research data. Abnormal coagulation marker monitoring occurs due to a lack of consistency in follow-up testing intervals and their implications on long-term patient survival [28]. Many studies pointed to improved results from coagulation marker-guided operations, yet these findings exist at a preliminary stage [29,30]. The available research illustrated encouraging proof regarding the utilization of coagulation markers for TIC assessment and management, but clinical implementation continued to encounter multiple hurdles. Future studies in this field must solve three fundamental problems, which include standardizing biomarker measurements alongside proper methodology control and collection of adequate data points. Multi-center research with larger study populations must be conducted to prove the reliability of biomarker tests for clinical outcome prediction within different trauma patient sets. Additional research must focus on how genetic components, along with molecular signaling pathways, influence TIC as part of determining the fundamental coagulation mechanism in traumatic conditions. Multiple prospective studies, together with clinical trials, need to be conducted to diagnose and guide therapy using coagulation marker tests in TIC.

## Conclusions

The analysis validates fibrinogen, thrombin, and APC as diagnostic blood markers that help identify and determine the outcomes of TIC. The severity of trauma injuries, along with mortality rates, increases and leads to worse clinical results when these biomarkers are present at elevated levels. The present studies have major limitations because of methodological inconsistencies, along with restricted sample size and varied follow-up periods, which hinder the application of these research results to a wide trauma population.

Upcoming studies involving large trauma patient populations need to validate how biomarkers help trauma care staff make improved treatment decisions and forecast the medical outcomes of their patients. The combination of genetic and molecular techniques will help explain blood clotting mechanisms better and create better methods to forecast the outcomes of trauma patients.
